# Phylogenetic diversity shapes salt tolerance in *Phragmites australis* estuarine populations in East China

**DOI:** 10.1038/s41598-020-74727-0

**Published:** 2020-10-19

**Authors:** Carla Lambertini, Wen-Yong Guo, Siyuan Ye, Franziska Eller, Xiao Guo, Xiu-Zhen Li, Brian K. Sorrell, Maria Speranza, Hans Brix

**Affiliations:** 1 Department of Biology, Aarhus University, Ole Worms Allé 1, 8000 Aarhus C, Denmark; 2 Laboratory for Marine Geology, Qingdao National Laboratory for Marine Science and Technology, Qingdao, 266061 People’s Republic of China; 3 Key Laboratory of Coastal Wetlands Biogeosciences, Qingdao Institute of Marine Geology, China Geologic Survey, Qingdao, 266061 People’s Republic of China; 4 College of Landscape Architecture and Forestry, Qingdao Agricultural University, Qingdao, 266109 People’s Republic of China; 5 State Key Laboratory of Estuarine and Coastal Research, East China Normal University, Shanghai, 200062 People’s Republic of China; 6 Department of Agricultural and Food Sciences, University of Bologna, 40127 Bologna, Italy

**Keywords:** Ecology, Evolution, Genetics

## Abstract

Estuaries are dynamic and selective environments that provide frequent opportunities for the turnover of *Phragmites australis* populations. We studied *Phragmites* genetic diversity patterns in three of the major deltas of China, viz*.* the Yellow River, the Yangtze and the Liaohe, in relation to *Phragmites* global phylogeography and soil salinity. We found that two distantly related *P. australis* haplotypes, each with intercontinental distribution, co-occur in these deltas in China. One is European *Phragmites* (Haplotype O) and is related to *P. japonicus*; the other (Haplotype P) has its range in East Asia and Australia and is related to the Asian tropical species *P. karka*. The two haplotypes have differing salt tolerance, with Haplotype O in areas with the highest salinity and Haplotype P in areas with the lowest. Introgressed hybrids of Haplotype P with *P. karka,* and F1 hybrids with Haplotype O, have higher salt tolerance than Haplotype P. Phylogenetic diversity appears as the factor that better explains population structure and salinity tolerance in these estuaries. Future research may explain whether the two *P. australis* haplotypes evolved in East Asia, and East Asia is a center of *Phragmites* diversity, or are introduced and a threat to *P. japonicus* and *P. karka*.

## Introduction

Estuaries are dynamic environments with frequent disturbance and extreme conditions, which provide repeated opportunities for the decline and the establishment of new populations. Propagules can be recruited from nearby populations or be introduced from distant and different environments by migratory birds, or international ship traffic. One of the most common and productive aquatic plants inhabiting the world’s estuaries is *Phragmites australis* (Cav.) Trin. ex Steud., a cosmopolitan tall grass with high intraspecific variation and ecological amplitude^1^. *Phragmites australis* populations are extremely phylogenetically diverse in the Mississippi River Delta^2,3^ and in the Danube Delta^4^. Phylogenetic variation, including ploidy variation^2–4^, provides fitness in these dynamic environments due to the enlarged eco-physiological adaptability provided by a gene pool of genotypes of multiple distinct origins^5–7^. Due to their genetic diversity and the strong and variable selection pressure of the environment, estuarine marshlands are evolutionary hotspots of *Phragmites* diversity and can be dangerous gateways for invasions, especially cryptic invasions, in ranges where *P. australis* is native. For example, *P. australis* invasion in North America started at New York Harbor in marshes of the Hudson River estuary, where introduced European *P. australis* displaced the native populations of *Phragmites australis* ssp. *americanus*^8,9^.

Salt tolerance is a competitive trait in brackish estuarine environments. As a species, *P. australis* tolerates a wide salinity range^10–14^, but genotypes of different phylogeographic origin differ in salinity tolerance^6,15^ due to their different bioclimatic origins. For example, the invasive Mediterranean *Phragmites* lineage thrives in the brackish Mississippi River Delta thanks to its adaptation to draught evolved in the warm, dry climate of its native range in the Mediterranean and Middle East^5,7^. In the Yellow River Delta in China the genetic diversity of *P. australis* decreases with increasing soil salinity and only specific allelic phenotypes occur in the saltiest patches of the mosaic of saline habitats in the delta^16^. Such distinctive genotypes may have evolved locally from native populations as well as from introduced pre-adapted genotypes.

In this study we investigate phylogenetic diversity and its role in salt adaptation in three estuarine *P. australis* populations in China, in the Yangtze, Yellow River and Liaohe deltas. Unlike previous local studies of *P. australis* diversity in Chinese coastal marshes, we analysed the genetic diversity patterns of the Chinese populations within the global phylogeographic structure of the genus *Phragmites*^17,18^ in order to resolve their phylogeographic relationships and trace possible introductions. Several *Phragmites* lineages have recently been identified in China and Korea^19–21^ and classified according to the *Phragmites* classification system introduced by Saltonstall based on chloroplast DNA sequences^9,22^, however a macro-perspective of the evolutionary history of the exceptionally high diversity of *Phragmites* lineages in East Asia is lacking. There are at least three *Phragmites* species in East Asia: *P. japonicus* Steud.*, P. karka* (Retz.) Trin. ex Steud.and *P. australis*, with ploidy variation from 2n = 4 × to 10x (with 2n = 8 × being dominant^23,24^), and several salt-tolerant ecotypes^10,16,25^. Four *P. australis* ecotypes have been phenotyped in the Yellow River Delta based on tissue Na^+^/K^+^ ratios as an indicator of salt stress^10^. Takahashi et al.^25^ showed that salt-tolerant reeds from the Yellow River Delta contained low Na^+^ and high K^+^ content compared to salt-sensitive plants from a freshwater river in Japan which contained high Na^+^ and low K^+^ when exposed to salt. This suggests that salt tolerant ecotypes in the Yellow River may be able to select K^+^ over Na^+^ in saline conditions and maintain ion homeostasis^25^. The *HKT1* gene functions as a K^+^/Na^+^ co-transporter in *P. australis* and an insertion in an *HKT1* intron causes alternative splicing of the mRNA into the two transcripts of the salt-tolerant and salt-sensitive reeds^25^. Variation in the genomic sequence of the *HKT1* gene, as documented by^25^ and by^20^ in wild Korean populations, may therefore distinguish salt-adapted and non-adapted genotypes^25^. Therefore, in addition to (1) the phylogenetic relationships, we investigated also the (2) genetic structure of *Phragmites* populations (inferred by SSR—or microsatellites) in relation to soil salinity, (3) the *HKT1* gene polymorphism and (4) the phylogenetic diversity of chloroplast DNA. Based on previous studies of estuarine *Phragmites* populations elsewhere, we expected high phylogenetic diversity in the study area and a salt-dependent distribution of the phylo-types of different origin.

## Results

### Phylogenetic relationships of the estuarine populations

**Figure 1:**
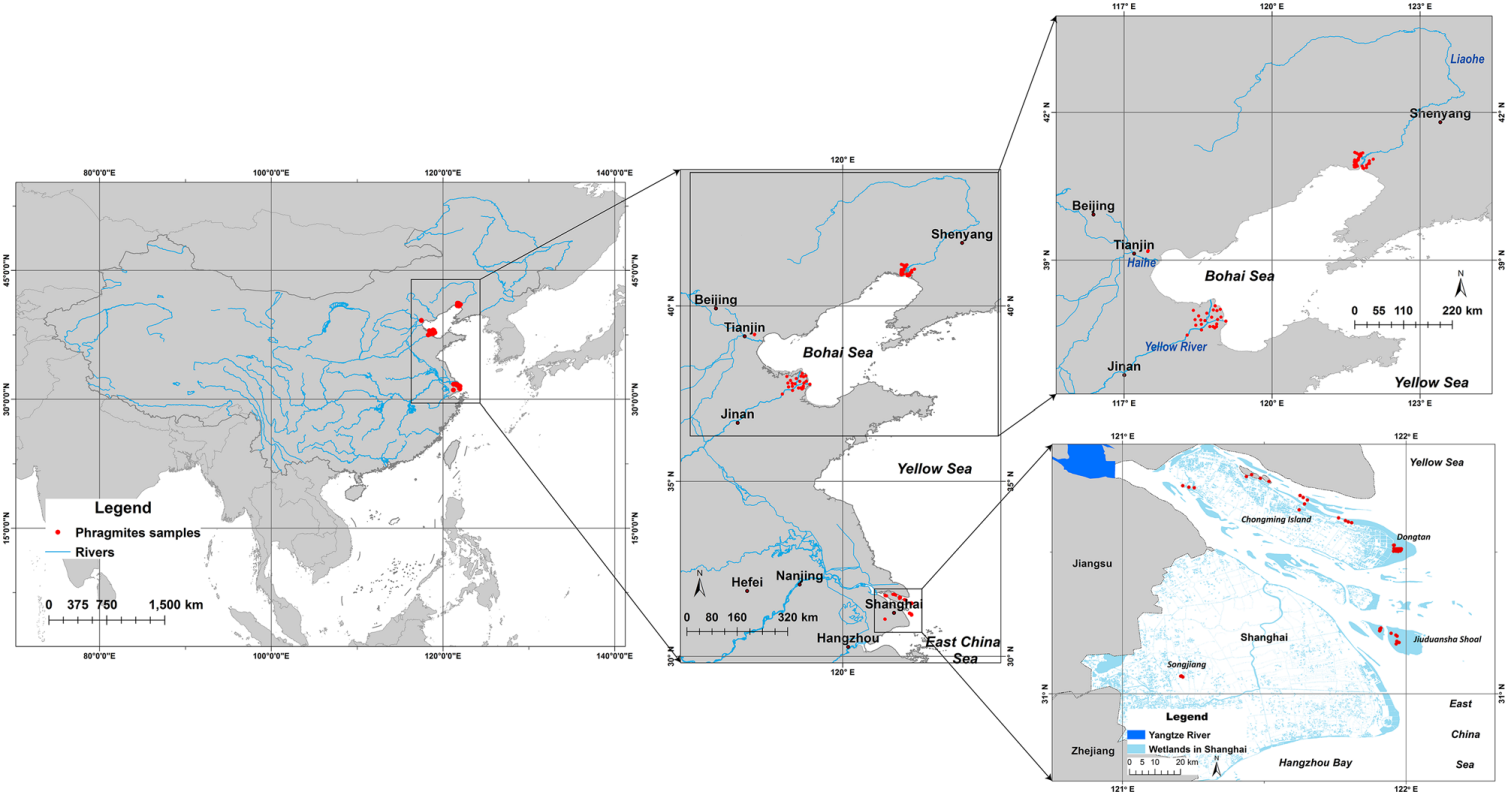
Map of the Chinese populations sampled in the study (ArcGIS ver. 10.6, Environmental Systems Research Institute, ESRI).

**Figure 2:**
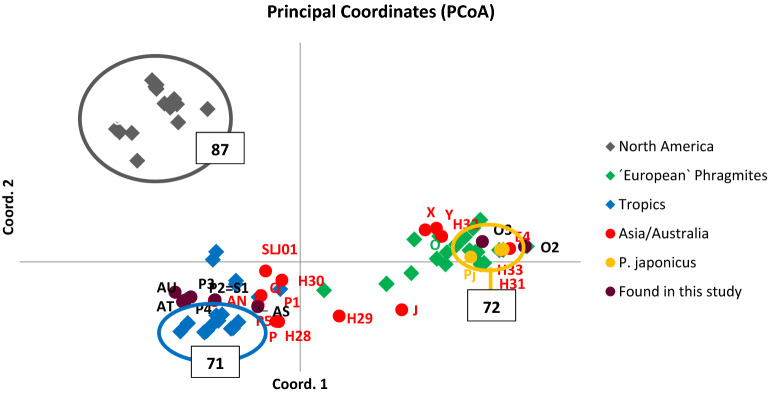
Phylogenetic position of the Chinese populations (purple) in the global phylogeographic structure of the genus *Phragmites,* inferred by cpDNA sequences (*trnT-trnL* and *rbcL-psaI*). The haplotypes previously identified in East Asia and Australia are in red. The coordinates account for 44% of the variation in the data (27% coord. 1 and 17% coord. 2). The circles with the numbers indicate the statistical support from the parsimony analiysis. Haplotypes IDs follow the names in GenBank. Haplotypes source: H28, H29, H30, H31, H32 and H33 (An et al. 2012). E4, S2 (corresponding to haplotype P1 in our study) and *Phragmites japonicus* (labeled *Pj*) (Chu et al., 2011). SLJ01 (Hurry et al. 2013). J, O, P, Q, X, Y (Saltonstall 2002). AN (Lambertini et al. unpublished). P1, P2, P3, P4, P5 and O2, O3 have been classified as microsatellites variants of haplotype P and O, as they differ from these in the numbers of repeats at the microsatellite loci. Statistical support is from the parsimony analysis. The PcoA is made with the program GenAlex ver. 6.4^45^.

Fragment size polymorphism in the *TrnT-TrnL short* region of the cpDNA revealed two haplotype profiles within the Chinese estuarine populations (Fig. 1, Supplementary Information S1). Following *Phragmites* classification system, the sequences matched *P. australis* Haplotype P and O and their intrahaplotypic microsatellite variants (P1, P2, P3, P4 and O2, O3) or differed from Haplotype P in one to two substitutions (new Haplotypes AS, AT, AU) (GenBank accessions no. KP994324 to KP994334). In total we found 9 different haplotypes of two distantly related phylogeographic groups (Fig. 2) previously defined as the Far East-Australian group (hereafter P-related genotypes) and “European” *Phragmites*, despite its almost cosmopolitan distribution (hereafter O-related genotypes). Compared to the previous phylogenies the inclusion of the new haplotypes from Asia and Australia moved the Asian/Australian group closer to the tropical species *P. mauritianus* and *P. karka* (note the haplotypes of *P. karka* marked blue in the lower part of the PCoA with 71% support, Fig. 2). The populations in the Yangtze River Delta (CML, JDSL, CXL and SJL) were entirely composed of Haplotype P-related genotypes, whereas the population in the Yellow River Delta consisted of O-related genotypes (with the exception of one single P-related genotype). The Tianjin population consisted of O-related genotypes and the Liaohe population was mixed with O- and P-related genotypes co-occurring in close proximity (Table S1).

**Figure 3:**
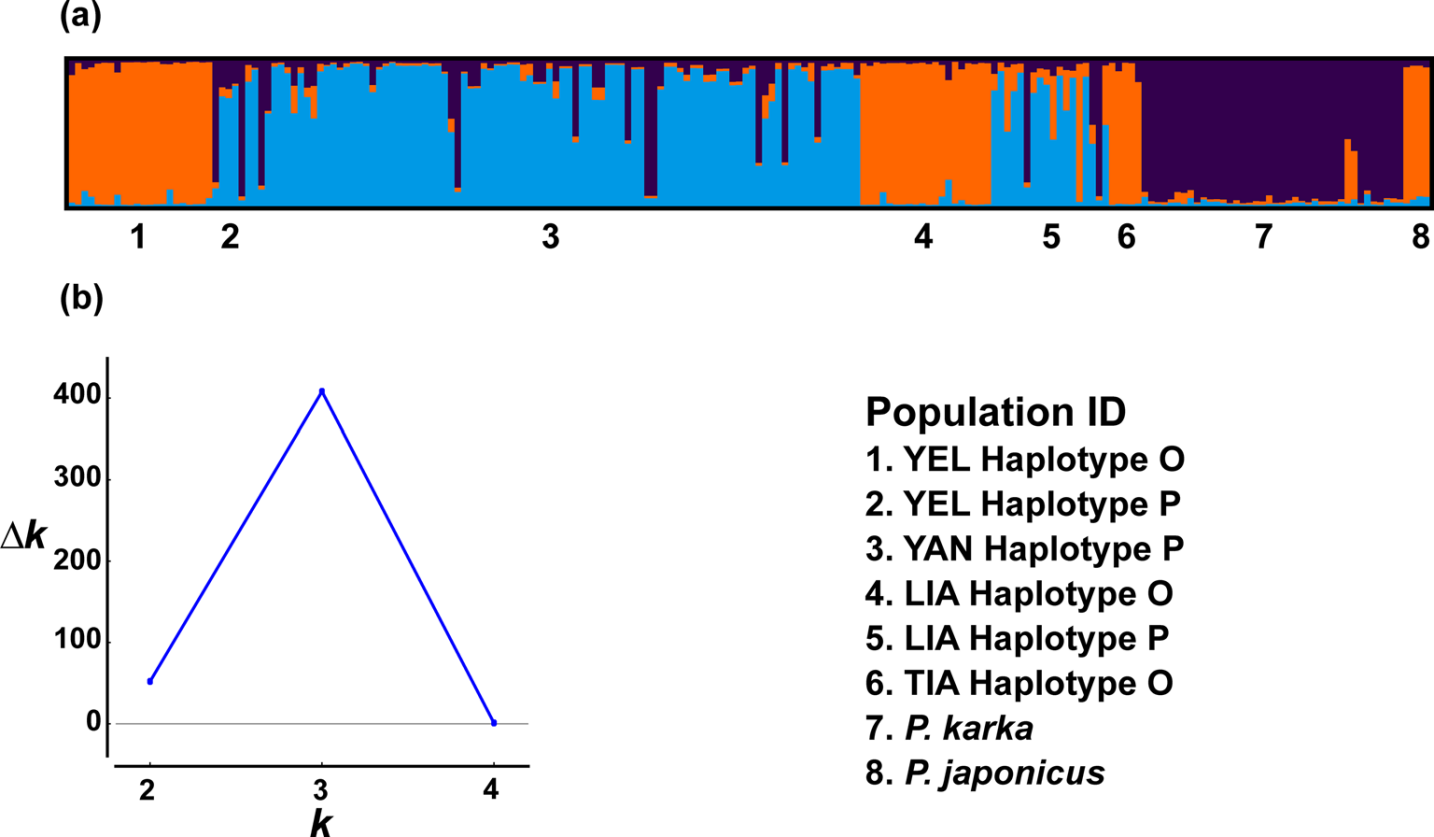
Population structure of SSR data. Inferred ancestry probability is in Y axis and population ID in X axis. (**a**) Population structure for K = 3. (**b**) Evanno’s “deltaK” inferring the most likely number of ancestral populations (K). The graph is made with Structure ver. 2.3.4^47^ and Evanno’s delta K graph is made with Structure Harvester^48^.

The Bayesian analysis of SSR data inferred three ancestral populations for the Chinese estuarine populations (Fig. 3): one for the Haplotype-O related genotypes and *P. japonicus* (Haplotype AM) (orange), one for the Haplotype P-related genotypes (light blue), and one for the hybrids of Haplotype P and *P. karka* (dark blue). This analysis revealed several hybrids in the Yangtze Delta populations between *P. australis* P-related genotypes and a population of *P. karka* in the Mekong Delta in Viet Nam used as a *P. karka* reference in this study (Haplotypes I and U and their microsatellite variants), and in the Liaohe Delta population between *P. australis* Haplotypes P- and O- related genotypes and between *P. australis* Haplotype P and *P. karka*. Both hybrid types were Haplotype P. Only one hybrid in the Liaohe Delta population was Haplotype O and had admixed ancestry (higher than 20%) with both Haplotype P and *P. karka* (Fig. 3).

### Population genetic structure

**Figure 4:**
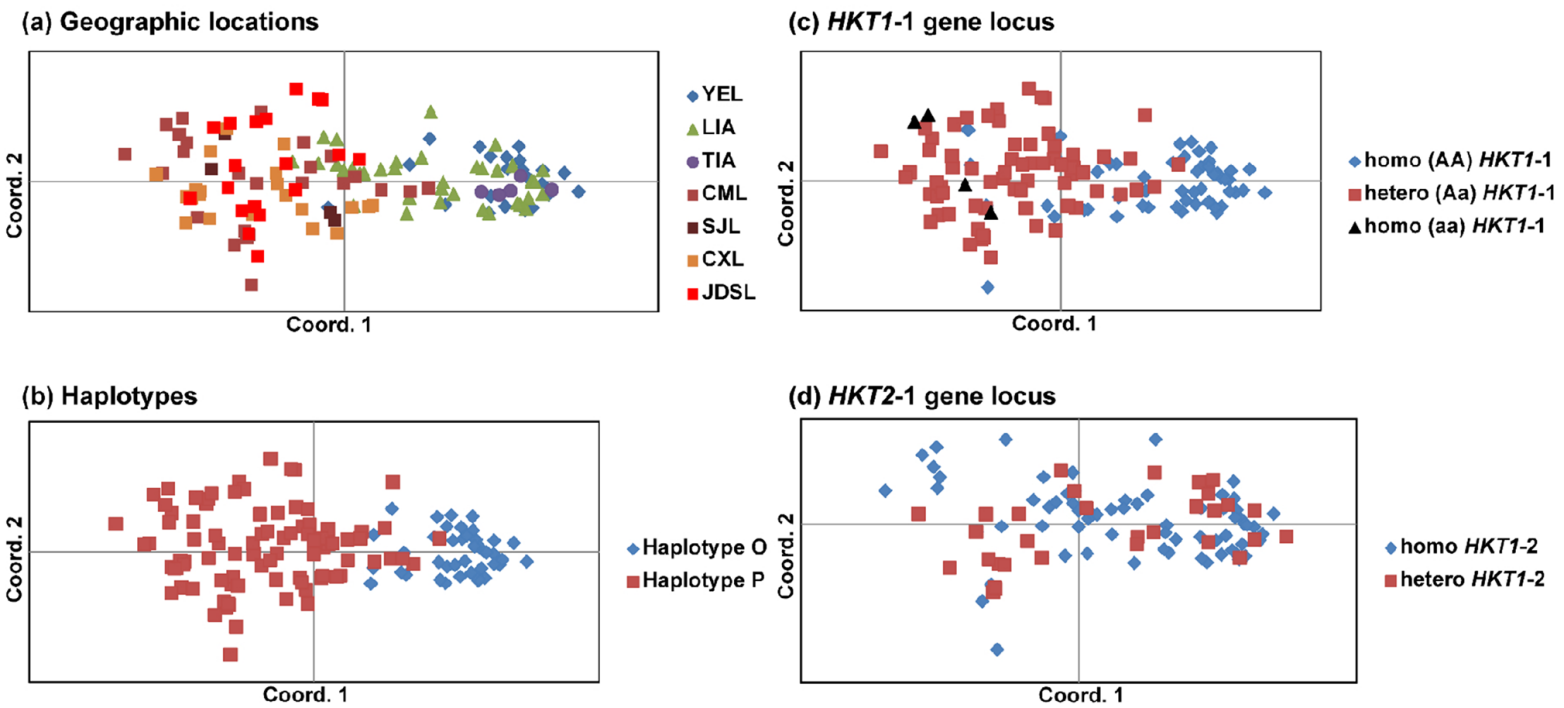
Principal coordinate analysis of SSR pairwise genetic distances. The coordinates account for 37% of the variation (14% coord. 1 and 9% coord. 2). (**a**) the different colours indicate genotypes of populations of different geographic locations. (**b**) The different colours indicate genotypes related to Haplotype O and Haplotype P inferred by the *trnT* short fragment. (**c**) The different colours indicate homozygotes and heterozygotes at the *HKT1*-1 gene locus. (**d**) The different colours indicate homozygotes and heterozygotes at the *HKT1*-2 gene locus. The PcoAs are made with the program GenAlex ver. 6.4^45^.

The SSRs divided the sample set into two main groups, which reflected the haplotypic structure of the populations better than their geographic distribution (Fig. 4a,b), and the structure of *HKT1* homo-and heterozygotes (Fig. 4c,d).

Significant genetic divergence among the seven populations (Supplementary Information S1) was detected by the AMOVA of SSR data (Table 1a). The extent of divergence increased when the variance was tested between Haplotype O- and P- related genotypes (Table 1b) and disappeared among the populations related to Haplotype O when population structure was tested within haplotypes (Table 1c) and within population (Table 1d). It remained significant among the populations related to Haplotype P, both among populations of different deltas (Table 1e) and among the populations in the Yangtze Delta (CML, CXL and JDSL) (Table 1f). Differences in genetic variance due to SSR alleles were also detected between homozygotes and heterozygotes at the *HKT1*-1 locus of the *HKT1* gene (Table 1g) (but not at the *HKT1*-2 locus, Table 1h), however such a distinction disappeared when the variance was tested between homo- and heterozygotes of Haplotype O (Table 1i,j). It remained significant between the homo- and heterozygotes of the Haplotype P-related genotypes both at the *HKT1*-1 and *HKT1*-2 loci (Table 1k,l).

**Table 1:** AMOVA of SSR data testing the structure within and among populations based on (1a) the geographic distribution of the populations, (2b–f) the haplotypic composition of the populations and (2g–l) the HKT1 composition of the populations in homo- and heterozygotes.

Analysis	Structure tested		N	Source of variation	df	SS	MS	Est Var	%	PhiPT	P-value
Ref	Regions	Pops									
(a)		YEL	23	Among pops	6	130,004	21,667	0.785	16%	0.161	0.000
		CML	34	Within pops	157	644,271	4,104	4,104	84%		
		SJL	6	Total	163	774,274		4,889	100%		
		CXL	32								
		JDSL	26								
		LIA	38								
		TIA	5								
(b)		HAPL P	117	Among pops	1	86,535	86,535	1,227	22%	0.224	0.000
		HAPL O	47	Within pops	162	687,740	4,245	4,245	78%		
				Total	163	774,274		5,472	100%		
(c)	HAPL O:	YEL	22	Among pops	2	6,021	3,010	0.049	2%	0.020	0.134
		LIA	20	Within pops	44	102,873	2,338	2,338	98%		
		TIA	5	Total	46	108,894		2,387	100%		
(d)	YEL,	Lobe 1	3	Among pops	6	13,045	2,174	0.000	0%	− 0.051	0.862
	HAPL O:	Lobe 2	4	Within pops	15	38,500	2,567	2,567	100%		
		Lobe 3	3	Total	21	51,545		2,567	100%		
		Lobe 4	3								
		Lobe 5	3								
		Lobe 6	3								
		Lobe 7	3								
(e)	HAPL P:			Among regions	1	22,982	22,982	0.413	8%	0.077	0.000
	YAN:	CML	36	Among pops within regions	3	33,970	11,323	0.292	5%	0.059	0.000
		SJL	6	Within pops	111	517,832	4,665	4,665	87%	0.131	0.000
		CXL	32	Total	115	574,784		5,369	100%		
		JDSL	26								
	LIA:	LIA	18								
(f)	YAN delta P	CML	34	Among regions	1	13,136	13,136	0.633	12%	0.118	0.002
		CXL	32	Among pops within regions	2	20,834	10,417	0.192	4%	0.040	0.000
		JDSL	26	Within pops	94	428,499	4,558	4,558	85%	0.153	0.000
	Yan urban P	SJL	6	Total	97	462,469		5,384	100%		
(g)	HKT1-1	Homo AA	68	Among pops	2	61,178	30,589	0.630	12%	0.124	0.000
		Hetero Aa	89	Within pops	158	701,269	4,438	4,438	88%		
		Homo aa	4	Total	160	762,447		5,069	100%		
(h)	HKT1-2	Homo BBB	131	Among pops	1	5,792	5,792	0.020	0%	0.004	0.204
		Hetero Bbc	33	Within Pops	162	768,482	4,744	4,744	100%		
				Total	163	774,274		4,764	100%		
(i)	HAPL O	Homo AA	34	Among pops	1	2,656	2,656	0.016	1%	0.007	0.317
	HKT1-1	Hetero Aa	13	Within pops	45	106,238	2,361	2,361	99%		
				Total	46	108,894		2,377	100%		
(j)	HAPL O	Homo BBB	34	Among pops	1	2,656	2,656	0.016	1%	0.007	0.317
	HKT1-2	Hetero Bbc	13	Within pops	45	106,238	2,361	2,361	99%		
				Total	46	108,894		2,377	100%		
(k)	HAPL P	Homo AA	23	Among pops	2	19,979	9,990	0.236	5%	0.046	0.000
	HKT1-1	Hetero Aa	89	Within pops	113	554,805	4,910	4,910	95%		
		Homo aa	4	Total	115	574,784		5,146	100%		
(l)	HAPL P	Homo BBB	97	Among pops	1	8,721	8,721	0.113	2%	0.022	0.017
	HKT1-2	Hetero Bbc	20	Within pops	115	570,125	4,958	4,958	98%		
				Total	116	578,846		5,071	100%		

*N* sample size, *df* degrees of freedom, *SS* Sum of squares, *MS* mean sum of squares, *Est. Var.* estimated variance, *%* percentage of estimated variance, *PhiPT* fixation index (analogue of Fst), P-value based on 9999 permutations.

Three to five alleles per locus were frequent in the SSR profiles of both the Liaohe and Yangtze Haplotype P-related genotypes, whereas Haplotype-O related genotypes showed a disomic inheritance pattern. However, two exceptions of genotypes with 3 alleles at locus Pagt4 were found also in the O- related populations in the Yellow River and Liaohe Deltas. Genetic diversity (I, h and uh) in SSRs was highest within the Haplotype-P related populations due to the higher number of alleles in P- related than in O-related genotypes (Table 2).

**Table 2:** Genetic diversity in haplotype O- and haplotype P-related populations.

Pop	N	N SE	Na	NA SE	Ne	Ne SE	I	SE I	h	h SE	uh	uh SE
YEL O	21.846	0.078	0.795	0.157	1.163	0.046	0.161	0.038	0.102	0.026	0.107	0.027
LIA O	19.795	0.075	0.795	0.157	1.149	0.039	0.159	0.036	0.099	0.024	0.104	0.025
TIA O	4.897	0.049	0.487	0.132	1.152	0.051	0.124	0.040	0.085	0.028	0.109	0.036
CML P	33.487	0.207	1.513	0.137	1.410	0.058	0.367	0.043	0.243	0.031	0.250	0.032
CXL P	31.487	0.197	1.333	0.153	1.312	0.053	0.302	0.041	0.194	0.029	0.201	0.030
JDSL P	25.692	0.117	1.231	0.158	1.331	0.057	0.305	0.044	0.200	0.030	0.208	0.032
LIA P	17.513	0.187	1.590	0.131	1.380	0.058	0.358	0.040	0.230	0.029	0.245	0.031
SJL P	6.000	0.000	0.744	0.150	1.245	0.058	0.203	0.047	0.140	0.033	0.168	0.039

*N* effective sample size, *Na* no. of different alleles, *Ne* no. of effective alleles, *I* Shannon Information Index, *h* gene diversity, *uh* unbiased gene diversity, *SE* standard error.

### Genetic diversity distribution in relation to soil salinity

**Figure 5:**
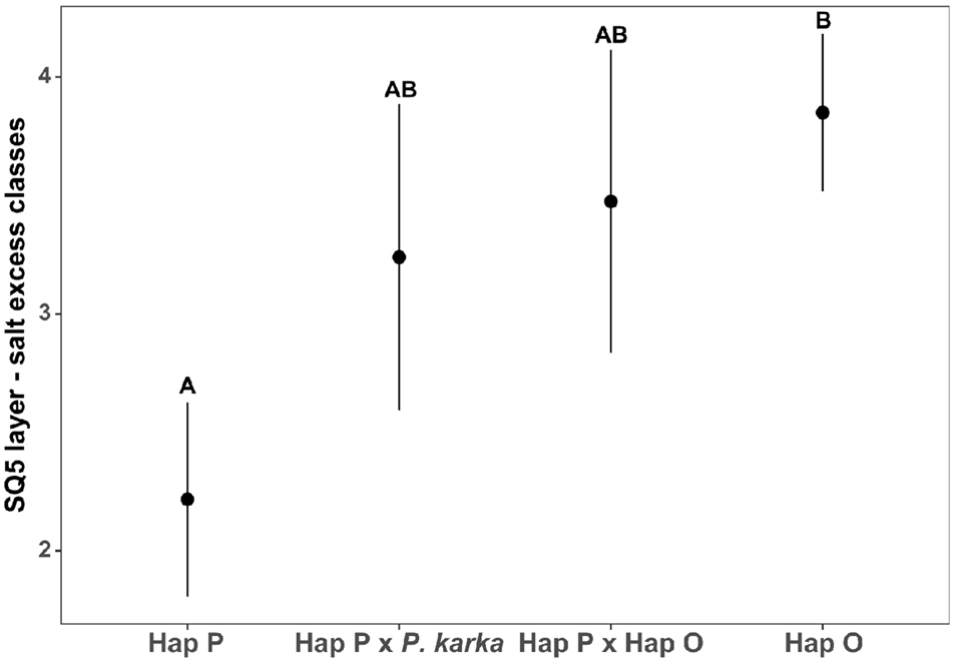
Distribution of phylo-types (X axis) by the salinity classes of the SQ5 layer of excess salt (Y axis). Average salt excess classes values and Bonferroni’s intervals for each phylo-type. (A) (AB) and (B) refer to the significance of the Bonferroni multiple comparison. The graph is produced in R (R core Team).

The factor that best explained the distribution of the genotypes in relation to salt was phylogenetic diversity, considering the four independent phylo-types: Haplotype O- and P-related genotypes and their hybrids (Haplotype P x *P. karka* and Haplotype P x Haplotype O) (*P* = 0.01). Genotypes related to Haplotype O were distributed in areas with higher salinity than those related to Haplotype P. Both hybrid types of Haplotype P had a higher variance in salt tolerance than Haplotype O- and P-related genotypes, and occurred in areas of intermediate salinity, compared to the two haplotypes (Fig. 5).

The distribution of *HKT1*-homozygotes and heterozygotes at both *HKT1-1* and *HKT1*-2 gene loci was not affected by salinity (*P* = 0.87 for *HKT1*-1; *P* = 0.50 for *HKT1*-2). The *HKT1* sequences of the homozygotes showed large variation, especially among the genotypes of Haplotype O. The sequences of the previously documented salt-tolerant genotype from Nanpi in the Yellow River Delta in GenBank matched the sequence of some of our Haplotype O genotypes in the Yellow River Delta. The sequences of the salt-sensitive genotype from Ustonia in Japan (also in GenBank) matched instead the sequences of our *P. japonicus* references in South Korea and Sakhalin Island (Russia), and was different from the sequences of Haplotype P-related genotypes in the Yangtze River Delta. The sequences of *P. karka* genotypes were different from those of *P. australis* Haplotype O and Haplotype P, as well as from those of *P. japonicus*. We found correlations neither between SSR allelic frequencies and the salinity classes of the Soil Quality layer 5 (SQ5) of the Harmonized World Soil Database, nor with our salinity measurements in the Yangtze Delta, nor between allelic and *HKT1* homo/heterozygotes frequencies, nor between *HKT1* homo/heterozygotes frequencies and salinity.

## Discussion

### Phylogenetic relationships of the estuarine populations

Haplotypes of two phylogeographic regions of *P. australis* co-occur in the coastal populations in East China. The genotypes related to Haplotype O are closely related to the European populations of *P. australis* and the East Asian endemic species *P. japonicus*, whereas Haplotypes P, AS, AT, AU are closely related to populations of *P. australis* in Australia. In this study they appeared closely related also to the tropical species *P. karka* in Asia and *P. mauritianus* in Africa. Haplotypes O and P are the dominant haplotypes in the studied populations and both have a wide distribution range. Halpotype O is found across temperate Europe^9,18^ and Asia^19^ and its cp-microsatellite variant O2 found in this study is shared by the two continents. Haplotype P has previously been found in East Asia and Australia^9,10,15,18,19^ and its cp-microsatellite P4 was also found in both continents. The other novel Haplotypes AS, AT and AU appeared to have evolved locally from Haplotype P- related genotypes, given their restricted ranges within the studied populations and the very few base substitutions compared to the sequences of Haplotype P-genotypes analysed in this study. Two populations were identified also by Gao et al.^16^ to explain the structure of the Yellow River population, and two ancestral populations were discovered for Korean *Phragmites*^20^. Gao et al.^16^ justified the two populations as salt-adapted and non-adapted genotypes, while Chu et al.^20^ explained the two ancestries as due to two *Phragmites* species, *P. australis* (sequenced as Haplotype P in that study) and *P. japonicus* in Korea. Two branches for *Phragmites australis* in East Asia were detected also by Yao et al.^26^ by ITS markers, that were identified as a suitable DNA barcode to capture the high genetic diversity of halo-tolerant Poaceae species in coastal areas.

In our study, Haplotype O-related genotypes occurred in areas with the highest salt impact and Haplotype P-related genotypes in areas with the lowest. As our samples from the Yellow River Delta were all Haplotype O-related genotypes, except for a single Haplotype P, and given our different geographic sampling from that of^16^, we cannot relate our results to those of their study directly. However, we also found a salt-dependent distribution of our samples that seems to be explained by genotypes of different phylogenetic origin which, in agreement with^16^, have distinct allelic patterns. In agreement with Chu et al.^20^, we found two distinct populations for *P. japonicus* and *P. australis* Haplotype P. As in our previous studies, *P. japonicus* is closely related to *P. australis* Haplotype O-related genotypes, both in cpDNA sequences and SSRs alleles^18^. In the present study Haplotype P appeared distantly related to its conspecific Haplotype O and more closely related to *P. karka*, with which it is also introgressed. *Phragmites karka* is known to occur and hybridize with *P. australis* haplotype P in southwestern China^21^. However, in contrast to^21^, *P. australis* Haplotype P is the seed donor of the hybrids that we found in the Yangtze and Liaohe deltas. Given the high degree of introgression and the lack of *P. karka* in our samples from the populations where the hybrids occur, *P. karka* introgression appears an inherited trait of Haplotype P rather than a recent local hybridization event.

The polysomic profiles of the SSRs alleles indicate a higher ploidy level for Haplotype P-related genotypes that consistently had more than two alleles, than that of Haplotype O-related genotypes that, instead, had a disomic pattern. Several studies have shown that Asian *P. australis* is dominated by octoploids and to a minor extent by hexaploids and decaploids, and that tetraploids are less frequent^17,23,24^. Based on our studies^17,18^ that sequenced the samples previously analysed for genome size by^24^, and our unpublished chromosome counts of Haplotype P and Haplotype O genotypes by “squash and stain”, Haplotype P-related genotypes are more frequently octoploids, whereas Haplotype O-related genotypes are more likely tetraploids, according to the sample sets that we studied.

The higher genetic diversity of Haplotype P octoploid populations (about the double of that of Haplotype O), and the high degree of introgression, suggests that Haplotype P is an allopolyploid that originated by hybridization between *P. karka* and another still unknown population different from Haplotype O and *P. japonicus*. Haplotype P shares in fact a large part of its genome with *P. karka*, but not with *P. australis* Haplotype O or *P. japonicus*, and the cpDNA is closely related to that of *P. karka*. Ishi and Kadono^27^ confirmed that octoploid *P. australis* is fertile in Asia. They found anomalies in neither pollen viability, nor availability, nor barriers to cross-pollination, nor reduced seed set rates in wild octoploid populations in Japan. The high genetic diversity that we found in our Haplotype P populations and the occurrence of hybrids between Haplotype P and Haplotype O in the Liaohe Delta further confirm that Haplotype P-related genotypes can reproduce sexually, and indicate that ploidy level is not a barrier to gene flow between the two Haplotypes. All hybrids between Haplotype P- and Haplotype O- related genotypes, except one, had the cpDNA of Haplotype P, indicating a higher ability of the octoploid genome to be recombined, but also a recent contact between Haplotypes P and O, as obvious hybrids were found only in the Liaohe populations where genotypes of the two lineages coexist. The Liaohe delta is one of the largest reed stands in the world and is managed for pulp and paper production^28^. Haplotype P could have been introduced here, and this could explain the co-dominance of the two distantly related haplotypes only in this delta.

The genetic diversity of Haplotype O-related populations was also relatively high and comparable to that of the European *P. australis* tetraploid population and higher than the diversity of the invasive European populations in North America and in the Gulf Coast^3^. Although this can rule out founder effects due to a recent introduction of Haplotype O to East Asia, the sympatry of the two distantly *P. australis* related lineages and in the same habitat, and their relationships to the two Asian species *P. karka* and *P. japonicus*, pose several questions concerning *P. australis* evolution in East Asia that cannot be explained by natural selection only. These results have therefore implications also for *Phragmites* taxonomy, systematics and ecology in East Asia and are central for future research.

### Population genetic structure

The populations of the two haplotypes also differed in genetic structure. Insignificant population differentiation measures (PhiPTs) confirm gene flow and long-distance dispersal of pollen and seeds among the populations related to Haplotype O, whereas the significant PhiPTs among the populations related to Haplotype P indicate differentiation among populations even at the local scale, as seen among the populations in the Yangtze Delta. Nevertheless, the low PhiPT values (max 0.16) support long-distance gene flow even among the Haplotype P-related populations. This significant structure could be due to the high genetic diversity dispersed by the seeds of an octoploid allopolyploid. Lack of a regional geographic structure is a common feature of *P. australis* populations in Europe^29–31^, North America^32^ and South Africa^33^. Even in Australia, Haplotype P populations are not genetically distinct^15^. The significantly different genetic variation pattern of Haplotype P populations in East Asia is therefore unusual for *P. australis*. Previous studies of *P. australis* intraspecific variation in China have found similar patterns and attributed the spatial differentiation to edaphic factors in the Songnen Prairie^34,35^, swamp, salt and dune reed ecotypes in the Hexi Corridor in the Gansu province^36^, and salt tolerance in the Yellow River Delta^11,16^ . None of these studies, however, resolved or considered the phylogenetic diversity of the populations, or realized that population structure and ecology are different within lineages. Resolving phylogenetic relationships appears therefore crucial from this study to address ecology and adaptation in future *Phragmites* studies in East Asia and elsewhere.

### Genetic diversity distribution in relation to salinity

The *HKT1* gene encodes one of the main mechanisms of salt tolerance in vascular plants^37^. It varies in the genomic sequences of *P. australis* in East Asia^20,25^ and there is allelic variation associated with salt response in wild coastal populations of *Arabidopsis thaliana* in Northern Europe^38^. In our study salinity did not affect the distribution of homo- and heterozygotes at the *HKT1*-1 and *HKT1*-2 sites of the *HKT1* gene, but the factor that better explained the relationship between salinity and genetic diversity, as well as the *HKT1* variation pattern, was phylogenetic origin. Haplotype O-related genotypes occurred in areas with the highest salinity and Haplotype P-related genotypes in areas with the lowest. Both Haplotype P hybrid types (Haplotype P x *P. karka* and Haplotype P x Haplotype O) occurred in a wider range of salinity than Haplotypes O and P, and had higher average salt tolerance than Haplotype P. The *P. karka* population in the present study is from the Mekong Delta in Viet Nam, another estuarine area subject to increasing salinization^39^. Salt tolerance as a specific trait of *P. karka* remains poorly understood, but our results show that hybridization between estuarine populations of *P. karka,* and Haplotype O, increased salt tolerance in Haplotype P in East China.

Compared to^25^ sequences, the pattern of genetic variation of the complete *HKT1* gene sequence of the homozygotes appeared more variable and complex than that of two distinct sequences for salt-tolerant and salt-sensitive genotypes. As could be expected, the coding sequence of salt-sensitive Haplotype P was different from that of salt-tolerant *P. karka*. Likewise, the coding sequence of salt-tolerant Haplotype O (matching the salt-tolerant coding sequence of^25^) was distinct from that of its close relative *P. japonicus* (matching the salt-sensitive sequence of^25^), suggesting that salt tolerance has evolved multiple times and independently in East China. The sequences of the hybrids could not be resolved with certainty because of heterozygosity at several loci.

## Conclusions

As we have shown previously^5–7^, phylogenetic relationships play a crucial adaptive role for *Phragmites* populations. Two distantly related *P. australis* haplotypes dominate the estuarine populations in East China. One, Haplotype O, is related to European *Phragmites *sensu Lambertini et al.^18^, the other, Haplotype P, is highly introgressed with *P. karka* and has its distribution in East Asia and Australia. The two haplotypes have different salt tolerance, with Haplotype O occurring in the most saline areas in the deltas and Haplotype P in the lowest. Hybridization with haplotype O and introgression with estuarine populations of *P. karka* has increased salt tolerance in Haplotype P and expanded its distribution range to the most saline areas in the Yangtze and Liaohe deltas. Saline tolerance has therefore evolved multiple times and independently in Haplotype P and Haplotype O. Interestingly, salt-sensitive Haplotype P is closely related to salt-tolerant Asian *P. karka*, whereas salt-tolerant Haplotype O is closely related to Asian *P. japonicus*, whose *HKT1* sequences matched those of the salt-sensitive genotypes of^25^. Future research may address the relationships of the two *P. australis* haplotypes with the two Asian species as well as the evolutionary and ecological significance of the two *P. australis* haplotypes in the range of the Asian species.

The present study has found no evidence of a cryptic invasion in East China, but cannot even resolve conclusively why two *P. australis* haplotypes, each with an intercontinental distribution range, do co-occur in the estuarine populations in East Asia and share the same habitat, i.e. whether East Asia is their center of diversity or they (one or both) have been introduced recently. The high genetic diversity in the populations of both haplotypes suggests an origin in East Asia, whereas the restricted distribution of their viable hybrids only to sympatry areas suggests a recent contact, or recent changes in the latitudinal distribution of the two haplotypes. The polysomic allelic pattern of Haplotype P further suggests a recent polyploidization. A larger sample of Haplotype O populations from across Europe and Asia and of Haplotype P from Australia and the Asian tropical region is needed to solve this enigma. Herbarium specimens may also help reconstruct past distribution ranges and can detect recent changes.

## Methods

### Sample set

We collected 174 samples of *P. australis* from 7 populations in East China in the Yangtze, Yellow River and Liaohe deltas and urban populations in the towns of Songjiang and Tianjin (Fig. 1). Accessions from outside of the area of investigation and of other *Phragmites* species were included to trace origins and relationships of the Chinese populations. In specific we used our sequence database^3,18^ and produced new sequences for *P. karka* and *P. japonicus* to cover the Asian *Phragmites* species variation. We used a *P. karka* population from the Mekong Delta in Viet Nam (N = 40) and samples of *P. japonicus* (N = 4) from populations in Korea and Sakhalin Island (Russia) (Supplementary Information S1).

### DNA extraction

DNA was extracted with the E.Z.N.A. tissue DNA kit (Omega Bio-tek Inc.) from apical leaves conserved in silica gel. The samples were ground in a mortar with quartz sand in liquid nitrogen before adding the E.Z.N.A. extraction buffer, following the protocol for dry plant specimens. The samples were treated with RNAase, eluted with 100 µl elution buffer, and conserved at -20 °C until amplification. DNA quality and quantity were checked in a Nano Drop Spectrophotometer ND-1000 (Saveen Werner) at 280 nm wavelength. DNA concentration exceeded 50 ng µl^−1^ in all samples.

### Chloroplast DNA markers

Two non-coding regions in the chloroplast DNA, the *trnT*-*trnL* and the *rbcL-psaI*, have previously been used to classify *Phragmites* diversity worldwide^3,9,18–20,22^. Of these two sequences, polymorphism in an indel of variable size in the *trnT*-*trnL* region can define most *Phragmites* haplotypes^41^. We used the primers of^41^ to amplify this variable region, and screened the polymorphism of our sample set. 2 µl DNA were added to 18 µl mastermix consisting of 10 µl 2xMastermix (VWR Amplicon), 10 pmol cy-labeled forward primer (5ʹ-CAT TAC AAA TGC GAT GCT CT – 3ʹ)^9^ , 10 pmol reverse primer (*TrnT*-R *short*: 5′- CGT CCG AGC CAT ATC AAA TT- 3ʹ) and sterile water to reach a final volume of 20 µl. Amplification was run in a Peltier Thermal Cycler PTC-200-MJ Research under the following conditions: 94 °C for 3 min, 40 cycles of 94 °C for 30 s, 52 °C for 40 s, 72 °C for 40 s, followed by 72 °C for 7 min. The amplified product (a fragment of about 350 bp) was diluted 20 × and loaded in a 7% acrylamide gel (Reprogel-Long REad) in the fragment analyser ALF Express II DNA Analysis System (Amersham Pharmacia Biotech). 5 µl of diluted PCR product were added to 3 µl loading dye (GE Healthcare) previously mixed with 1 µl each of 100 and 300 bp internal sizers (GE Healthcare). DNA was denaturated in the PTC-200 PCR at 94 °C for 5 min prior to loading in the gel. Electrophoresis conditions were 1500 V, 55 °C, 120 min. The first and the last slots of the gel were loaded with 50–500 bp external sizers (GE Healthcare).

All different *trnT*-*trnT*R*short* profiles were subsequently sequenced with the *trnT*-*trnL* and *rbcL-psaI* primers developed by^9^. Annealing temperatures were 50 °C for both primer sets and the PCR conditions were as for the fragments amplification except that the extension time was increased to 1 min. The PCR product was diluted 20 × and sent to Macrogen Korea for Sanger sequencing with forward and reverse primers in an ABI system.

We downloaded all *Phragmites trnT*-*trnL* and *rbcL-psaI* sequences from GenBank^22^. We aligned our sequences with those downloaded from GenBank with the program BioEdit ver. 7.0 Sequence Alignment Editor^40^. The initial “clustal” alignment was completed manually. Repeated motifs (minisatellites and microsatellites) and indels were coded as multistate characters. Sequences differing in the number of repeats at microsatellite loci were classified as cp-microsatellite variants following^18,22^. The final matrix of 134 sequences, 33 of which were from this study, and 1625 base pairs plus 27 multistate characters, was analysed with PAUP ver. 4.0b10^42^. The parsimony analysis was performed with 37% jackknife deletion, 1000 replicates, jack resampling and stepwise addition^43^. Individual pairwise genetic distances were also calculated as "total character difference". The matrix of genetic distances was imported in GenAlex ver. 6.4^44,45^ and analysed in a Principal Coordinate Analysis (PCoA). The PCoA obtained is comparable to that of Fig. 2 of^18^ and includes all haplotypes so far identified in East Asia.

We followed^9^ to define *Phragmites* Haplotypes and their intrahaplotypic microsatellites variants^18,22^ and^17,18^ for the names of the phylogeographic units.

### HKT1 gene polymorphism and gene sequences

We downloaded all *Phragmites* genomic and mRNA sequences of the Pha*HKT1* gene from GenBank^20,25^. We split the 2000 bp gene sequence into two fragments, *HKT1*-1 and *HKT1*-2, of about 1000 bp each. The *HKT1*-1 fragment contained an indel which was previously found to occur in the Yellow River population (Nanpi and Enchi) but not to have an obvious effect on the ability to tolerate salt, as both mRNA profiles occurred in salt-tolerant reeds^25^. The *HKT1*-2 fragment contained two introns, one of which was shown to splice and be translated into proteins of different size in salt-tolerant and salt-sensitive reeds^25^. We designed a set of primers around the indel in the *HKT1*-1 part of the gene and another set around the two introns in the *HKT1*-2 part of the gene, and analysed fragment size polymorphism.

The primers for the fragment containing the indel in the *HKT1*-1 part of the gene were *HKT1* Full-F2^25^ and *HKT1*-1R short (Supplementary Information S2) and annealing temperature was 55 °C. The primers for the fragment containing the two introns in the *HKT1*-2 part of the gene were *HKT1*-2F short and *HKT1*-2R End (Supplementary Information S2) and annealing temperature was 52 °C. Mastermix and PCR conditions were as described for the *trnT-TrnL* fragments. The amplified products (between 200 and 300 bp for the *HKT1*-1 fragments and around 400 bp for the *HKT1*-2 fragments) were run in a 1.5% agarose gel at 130 V for 2 to 5 h and band profiles scored manually.

We then sequenced all homozygote profiles. The *HKT1*-1 part of the gene was sequenced with the primers *HKT1* Full-F2^25^ and *HKT1*-1 1000R (Supplementary Information S2) and the annealing temperature was 60 °C. The *HKT1*-2 part of the gene was sequenced with the primers HTK1-2 1000F and *HKT1*R End (Supplementary Information S2) and the annealing temperature was 52 °C. Mastermix and PCR conditions were the same as described for the *trnT*-*trnL* and *rbcL-psaI* sequences. The amplified products were sequenced with the primers *HKT1* Full-F2, *HKT1*-2 1000F and *HKT1*R End.

We calculated the genotypic frequencies of homozygotes and heterozygotes at the two loci as the number of individuals sharing the same profile divided by the total number of individuals in the population. *HKT1*-1 had two alleles and three genotypic classes, whereas *HKT1*-2 had three alleles and only two genotypic classes (homozygotes and heterozygotes with three alleles; Table S3). We used a two-loci codominant matrix to calculate allelic frequencies with the program GenAlex.

The three sequences obtained for each homozygote genotype (*HKT1* Full-F2, *HKT1*-2 1000F and *HKT1*R End) were assembled with the program Genious ver. 6.1.7 (Biomatters) and the resulting consensus sequences of about 2000 bp were aligned with the same program. We compared our sequences, including those of *P. karka* and *P. japonicus*, with those of salt-tolerant and salt-sensitive ecotypes deposited in Gene Bank^25^ also included in the alignment.

### SSRs

We studied the allelic variation at loci PAGT4, PAGT8, PAGT9 and PAGT13 previously reported to vary in the Yellow River Delta in relation to soil salinity^16^ with the primers developed by^46^. Mastermix, PCR and electrophoresis conditions were the same as described for the *trnT-trnL* fragment size polymorphism. Annealing temperatures were 50 °C for PAGT 8, PAGT9 and PAGT13 and 54 °C for PAGT 4. The amplified product was diluted 20 × before loading into the fragment analyser.

The chromatograms were aligned with the ALFwin fragment analyser 1.00.36 (Amersham Pharmacia Biotech). Given the polysomic nature of the sample set and the co-occurrence of several ploidy levels in East Asia^24^, we scored for the presence/absence of alleles of different size. The final binary matrix included 174 taxa, 39 characters and 90 missing data, corresponding to 1.13% of the data, due to fragments of uncertain size.

A PCoA based on the pairwise Euclidean distances of binary haploid profiles was obtained with GenAlex and the resulting structure was investigated in relation to the geographic distribution of the populations, the haplotypic diversity inferred by the *TrnT* short fragment, and the homo- and heterozygotes at the *HKT1* gene loci. The significance of each factor (phylogenetic diversity, *HKT1* polymorphism) on the SSR genetic structure was tested with AMOVA (using GenAlex) and with the Bayesian clustering software Structure (ver. 2.3.4)^47^. Statistical significance for the AMOVA was obtained with 9999 permutations. Concerning Structure, we used the admixture model with correlated allelic frequencies to infer the number of ancestral populations, 300,000 burn-in periods, 1,000,000 MCMC reps after burn-in, and 20 iterations. The initial admixture coefficient alpha was set at 1.0^47^ and K was set from 1 to 10. Structure Harvester^48^ inferred the most likely K according to the Evanno´s method^49^.

Genetic diversity indices (samples size, number of alleles, number of effective alleles, Shannon Information Index, gene diversity and unbiased gene diversity) were calculated with GenAlex based on the frequency statistics of binary haploid profiles.

### Soil salinity

We used the Soil Quality layer 5 (SQ5) of the Harmonized World Soil Database v 1.2^50^ to extract salinity data at the coordinates of our samples. The SQ5 data file combines soil salinity with soil sodicity and soil phases influencing salt conditions and defines 7 categories of excess salt in the soil. The first five categories range from no to severe excess salt, while categories 6 and 7 include water bodies and permafrost areas. We compared the SQ5 layer with published soil salinity maps of the Yellow River Delta^16,51–53^ and of the Yangtze River Delta^54^ to ensure updated data and reproducibility of the SQ5 results. As some of our samples in the Yangtze River Delta were collected in areas that were still submersed in the SQ5 layer, we also mined salinity data from our own field work previously conducted at the sampling locations (Xiu Zhen-Li, unpublished data).

We tested differences in excess salt categories among haplotypes and among homo- and heterozygotes at the *HKT1*-1 and *HKT1*-2 loci in a multiple factor-ANOVA with Statgraphics Centurion, using General Linear Models and Bonferroni tests at the 95% confidence level. For the Yangtze River Delta populations, we repeated the ANOVA with salinity data measured in the field and also tested Pearson correlations between the SSR allelic frequencies and salinity (as per^16^), as well as between salinity and *HKT1*-1 and *HKT1*-2 allelic frequencies, and homo- and heterozygotes frequencies at the *HKT1*-1 and *HKT1*-2 loci, with Statgraphics Centurion.

## Supplementary information

Supplementary file1

## Data Availability

GenBank Accessions: KP994327 + KP994334 = Haplotype AS, KP994328 + KP994332 = Haplotype AT, KP994329 + KP994333 = Haplotype AU.

## References

[1] Eller F (2017). Cosmopolitan species as models for ecophysiological responses to global change: The common reed *Phragmites australis*. Front. Plant Sci..

[2] Hauber DP, Saltonstall K, White DA, Hood CS (2011). Genetic variation in the common reed, *Phragmites australis*, in the Mississippi River Delta marshes: Evidence for multiple introductions. Estuar. Coast.

[3] Lambertini C (2012). Tracing the origin of Gulf Coast *Phragmites* (Poaceae): A story of long-distance dispersal and hybridization. Am. J. Bot..

[4] Lambertini, C.*et al.* Revisiting *Phragmites australis*variation in the Danube Delta with DNA molecular techniques. *Water Resources and Wetlands, Conference Proceeding,* 142–150; https://www.limnology.ro/water2012/Proceedings/019.html (2012).

[5] Nguyen LX (2013). Are the photosynthetic characteristics of co-existing lineages of Phragmites australis based on adaptations acquired in their native climatic zones?. AoB Plants.

[6] Eller F, Lambertini C, Nguyen LX, Brix H (2014). Increased invasive potential of nonnative *Phragmites australis*: Elevated CO_2_ and temperature alleviate salinity effects on photosynthesis. Change Biol Glob.

[7] Guo W-Y (2016). Phenotypic traits of the Mediterranean *Phragmites australis* M1 lineage: Differences between the native and introduced ranges. Biol. Invasions.

[8] Chambers RM, Meyerson LA, Saltonstall K (1999). Expansion of *Phragmites australis* into tidal wetlands of North America. Aquat. Bot..

[9] Saltonstall K (2002). Cryptic invasion by a non-native genotype of the common reed, *Phragmites australis*, into North America. Proc. Natl. Acad. Sci. USA.

[10] Zhao KF, Feng LT, Zhang SQ (1999). Study of the salinity-adaptation physiology in different ecotypes of *Phragmites australis* in the Yellow River Delta of China: Osmotica and their contribution to the osmotic adjustment. Estuar. Coast Shelf.

[11] Guo W, Wang R, Zhou S, Zhang S, Zhang Z (2003). Genetic diversity and clonal structure of *Phragmites australis* in the Yellow River delta of China. Bio. Chem. Syst. Ecol..

[12] Vasquez EA, Glenn EP, Brown JJ, Guntensprenger GR, Nelson SG (2005). Salt tolerance underlies the cryptic invasion of North American salt marshes by an introduced haplotype of the common reed *Phragmites australis* (Poaceae). Mar. Ecol. Prog. Ser..

[13] Hurry CR, James EA, Thompson RM (2013). Connectivity, genetic structure and stress response of *Phragmites australis*: issues for restoration in a salinizing wetland system. Aquat. Bot..

[14] Holmes GD, Hall NE, Gendall AR, Boon PI, James EA (2016). Using transcriptomics to identify differential gene expression in response to salinity among Australian *Phragmites australis* clones. Front. Plant Sci..

[15] Achenbach L, Eller F, Nguyen LX, Brix H (2013). Differences in salinity tolerance in genetically distinct *Phragmites australis* clones. AoB Plants.

[16] Gao L (2012). Spatial genetic structure in natural populations of *Phragmites australis* in a mosaic of saline habitats in the Yellow River Delta China. PONE.

[17] Lambertini C (2006). A phylogeographic study of the cosmopolitan genus *Phragmites* (Poaceae) based on AFLPs. Plant. Syst. Evol..

[18] Lambertini C, Sorrell BK, Riis T, Olesen B, Brix H (2012). Exploring the borders of European *Phragmites* within a cosmopolitan genus. AoB Plants.

[19] An J, Wang Q, Yang J, Liu Q (2012). Phylogeographic analyses of *Phagmites australis* in China: Native distribution and habitat preference of the haplotype that invaded North America. J. Syst. Evol..

[20] Chu H (2011). Identification of natural hybrids in Korean *Phragmites* using haplotype and genotype analyses. Plant Syst. Evol..

[21] Tanaka T, Chagan I, Tatsuya I (2017). Phylogenetic analyses of Phragmites spp. in southwest China identified two lineages and their hybrids. Plant Sys. Evol..

[22] Saltonstall K (2016). The naming of *Phragmites* haplotypes. Biol. Invasions.

[23] Zong W, Chen R, Taniguchi K, Kondo K (1991). A chromosome study in intraspecific polyploidy of *Phragmites australis* and its related species. La Kromosomo.

[24] Clevering OA, Lissner J (1999). Taxonomy, chromosome numbers, clonal diversity and population dynamics of *Phragmites australis*. Aquat. Bot..

[25] Takahashi R, Liu S, Takano T (2007). Cloning and functional comparison of a high affinity K+ transporter gene Pha*HKT1* of salt-tolerant and salt-sensitive reed plants. J. Exp. Bot..

[26] Yao P-C (2017). Evaluating sampling strategy for DNA barcoding study of coastal and inland halo-tolerant Poaceae and Chenopodiaceae: A case study for increased sample size. PLoS ONE.

[27] Ishii J, Kadono Y (2002). Factors influencing seed production of *Phragmites australis*. Aquat. Bot..

[28] Brix H (2014). Large-scale management of common reed, *Phragmites australis*, for paper production: A case study from the Liaohe Delta, China. Ecol. Eng..

[29] Lambertini C, Gustafsson MHG, Frydenberg J, Speranza M, Brix H (2008). Genetic diversity patterns in *Phragmites australis* at the population, regional and continental scales. Aquat. Bot..

[30] Fér T, Hroudová Z (2009). Genetic diversity and dispersal of *Phragmites australis* in a small river system. Aquat. Bot..

[31] Krzakowa M, Michalak M (2010). Genetic differentiation of common reed (*Phragmites australis*) populations from selected lakes of Pomerania (NW Poland), revealed by elecrophoretically detected peroxidase variability. Biodivers. Resour. Conserv..

[32] McCormick M, Kettenring KM, Baron HM, Whigham D (2010). Spread of invasive *Phragmites australis* in estuaries with differing degrees of development: genetic patterns Allee effects and interpretation. J. Ecol..

[33] Canavan K, Paterson ID, Lambertini C, Hill MP (2018). Expansive reed populations—alien invasion or disturbed wetlands?. AoB PLANTS.

[34] Li M (2008). Clonal genetic diversity and populational genetic differentiation in *Phragmites australis* distributed in the Songnen Prairie in northeast China as revealed by amplified length polymorphism and sequence-specific amplification polymorphism molecular markers. Ann. Appl. Biol..

[35] Qiu T, Jiang LL, Yang YF (2016). Genetic and epigenetic diversity and structure of *Phragmites australis* from local habitats of the Songnen Prairie using amplified fragment length polymorphism markers. Genet. Mol. Res..

[36] Lin WF, Chen LJ, Zhu XY (2007). An analysis of genetic diversity of different ecotypes of reed (*Phragmites communis* Trin) by molecular marker techniques. J. Plant. Physiol. Mol. Biol..

[37] Rus A (2001). At*HKT1* is a salt tolerance determinant that controls Na^+^ entry into plant roots. Proc. Natl. Acad. Sci. USA.

[38] Baxter I (2010). A coastal cline in sodium accumulation in *Arabidopsis thaliana* is driven by natural variation of the sodium transporter At*HKT1*. PLoS Genet..

[39] Thuong VT (2019). Examining spatiotemporal salinity dynamics in the Mekong River Delta using Landsat time series imagery and a spatial regression approach. Sci. Tot. Environ..

[40] Hall TA (1999). Bioedit: A user-friendly biological sequence alignment editor and analysis program for Windows 95/98/NT. Nucleic Acids Symp. Ser..

[41] Lambertini C (2016). Heteroplasmy: Another complexity of the *Phragmites* genome to take into account. Biol. Invasions.

[42] Swofford, D.L. PAUP. Phylogenetic Analysis Using Parsimony (and other methods). Version 4. *Sinauer Associates*, Sunderland, Massachusetts (2002).

[43] Farris JS, Albert VA, Källersjö M, Lipscomb D, Kluge AG (1996). Parsimony jackknifing outperforms neighbor-joining. Cladistics.

[44] Peakall R, Smouse PE (2012). GenAlEx 6.5: genetic analysis in Excel. Population genetic software for teaching and research—an update. Bioinformatics.

[45] Peakall R, Smouse PE (2006). GenAlex 6: genetic analysis in Excel. Population genetic software for teaching and research. Mol. Ecol. Notes.

[46] Saltonstall K (2003). Microsatellite variation within and among North American lineages of *Phragmites australis*. Mol. Ecol..

[47] Pritchard KJ, Stephens M, Donnelly P (2000). Inference of population structure using multilocus genotype data. Genetics.

[48] Earl DA, von Holdt BM (2012). Structure Harvester: A website and program for visualizing STRUCTURE output and implementing the Evanno method. Conserv. Genet. Resour..

[49] Evanno G, Regnaut S, Goudet J (2005). Detecting the number of clusters of individuals using the software STRUCTURE: A simulation study. Mol. Ecol..

[50] Fischer, G. *et al.* Global agro-ecological zones assessment for agriculture (GAEZ 2008). *IIASA,* Laxenburg, Austria and FAO, Rome, Italy (2008)

[51] Lv ZZ (2013). Spatial variability of soil salinity in Bohai Sea coastal wetlands, China: Partition into four management zones. Plant Biosyst..

[52] Fan X, Liu Y, Tao J, Weng Y (2015). Soil salinity retrieval from advanced multi-spectral sensor with Partial Least Square Regression. Remote Sens..

[53] Yang L, Huang C, Liu G, Liu J, Zhu AX (2015). Mapping soil salinity using a similarity-based prediction approach: A case study in Huanghe River Delta China. Chin. Geogr. Sci..

[54] Jiang JY (2015). Soil organic carbon storage in tidal wetland and its relationships with soil physico-chemical factors: A case study of Dongtan of Chongming, Shanghai. J. Ecol. Rural Environ..

