# Supplementary material for: Phylogenetic diversity shapes salt tolerance in Phragmites australis estuarine populations in East China
Source: Sci Rep. 2020 Oct 19;10:17645. doi: 10.1038/s41598-020-74727-0 (PMC7572363; doi:10.1038/s41598-020-74727-0)
Supplement: Supplementary file 1 — Supplementary file1 [file 41598_2020_74727_MOESM1_ESM.docx]

Supplementary information

**Phylogenetic diversity shapes salt tolerance in *Phragmites* *australis* estuarine populations in East China**

Carla Lambertini* ^(1,6)^, Wen-Yong Guo ^(1)^, Suyen Ye ^(2,3)^, Franziska Eller ^(1)^, Xiao Guo ^(4)^, Xiu-Zhen Li ^(5)^, Brian K. Sorrell ^(1)^, Maria Speranza ^(6)^ and Hans Brix ^(1)^

^(1)^ Department of Biology, Aarhus University, Ole Worms Allé 1, Aarhus C, 8000, Denmark

^(2)^ Laboratory for Marine Geology, Qingdao National Laboratory for Marine Science and Technology, Qingdao, 266061, P. R. China

^(3)^ Key Laboratory of Coastal Wetlands Biogeosciences, Qingdao Institute of Marine Geology, China Geologic Survey, Qingdao, 266061, P. R. China

^(4)^ College of Landscape Architecture and Forestry, Qingdao Agricultural University, Qingdao 266109, P.R. China.

^(5)^ State Key Laboratory of Estuarine and Coastal Research, East China Normal University, Shanghai 200062, China

^(6)^ Department of Agricultural and Food Sciences, University of Bologna, Bologna 40127, Italy

* Corresponding author: carla.lambertini @unibo.it

**Table S1.** Sampling locations

| **Populations** | **Country** | **Pop ID** | **N** | **Description of sampling locations** |
| --- | --- | --- | --- | --- |
| ***P. australis* populations** |  |  |  |  |
| **Yangtze River Delta:** | China | YAN | 98 |  |
| Chongming Doungtan |  | CML | 34 | National Nature Reserve |
| Chongming Island |  | CXL | 32 | Farmlands |
| Jiuduansha Shoal |  | JDSL | 26 | New accreated island |
| Songjiang District |  | SJL | 6 | Town |
|  |  |  |  |  |
| **Yellow River delta** | China | YEL | 23 |  |
| Lobe 1 |  |  | 3 | Marsh |
| Lobe 2 |  |  | 4 | Marsh |
| Lobe 3 |  |  | 3 | Marsh |
| Lobe 4 |  |  | 3 | Marsh |
| Lobe 5 |  |  | 3 | Marsh |
| Lobe 6 |  |  | 3 | Marsh |
| Lobe 7 |  |  | 3 | Marsh |
|  |  |  |  |  |
| **Tianjin** | China | TIA | 5 |  |
| Dong Li Hu Lake |  |  |  | Town |
|  |  |  |  |  |
| **Liahoe River Delta** | China | LIA | 48 | Delta |
| ***P. japonicus* populations** |  | Pj | 4 |  |
| Gyeonggi-do | S. Korea |  | 1 | Suip River |
| Seul | S. Korea |  | 1 | Dorim River |
| Petrova, Sakhalin | Russia |  | 1 | Town |
| Lazovsky Reserve | Russia |  | 1 | Nature Reserve |
| ***P. karka* population** |  |  |  |  |
| Mekong Delta | Vietnam | Pk | 40 | Marsh |

Supplementary Information

**Table S2.** Primers designed to study the polymorphism in the HKT1 gene.

| **Primer name** | **Primer sequence (5´- 3´)** | **References** |
| --- | --- | --- |
| HKT1full-F2 | GAT CAG ATG GAG GCA TTC CA | Takahashi et al. 2007 |
| HKT1-1 R short | CGA GGG CTG AAG TCA GGG TT |  |
| HKT1 1000 R | GCT GAG CCC ATC GAA CAC CA |  |
| HKT1 1000 F | GCG GTT TCG TCA TCT GCT TC |  |
| HKT1-2 F short | CGA CAG AAA GCA GCT CAT GT |  |
| HKT1 end R | CAG GCA GTA CAC CAA TGA CA |  |

Supplementary information

**Table S3.** Genotypic frequencies (f) of homozygotes and heterozygotes at the HKT1-1 and HKT1-2 loci of the High Affinity K+ transport gene HKT1.

| **Structure** |  | **N** | **HTK1 -1** |  |  |  | **HKT1-2** |  |  |
| --- | --- | --- | --- | --- | --- | --- | --- | --- | --- |
|  |  |  | **f (AA)** | **f (Aa)** | **f (aa)** |  | **f (BBB)** | **f (BbC)** | |
| **Population** |  |  |  |  |  |  |  |  |  |
| **YEL** |  | **23** | **0.913** | **0.043** |  |  | **0.739** | **0.261** |  |
|  | YEL O | 22 | 0.955 | 0.455 |  |  | 0.727 | 0.273 |  |
|  | YEL P | 1 |  | 1.000 |  |  |  | 1.000 |  |
| **YAN** |  | **98** | **0.184** | **0.776** | **0.041** |  | **0.816** | **0.184** |  |
|  | CML P | 34 | 0.176 | 0.765 | 0.059 |  | 0.765 | 0.235 |  |
|  | SJL P | 6 | 0.667 | 0.333 |  |  | 1.000 |  |  |
|  | CXL P | 32 | 0.125 | 0.875 |  |  | 0.875 | 0.125 |  |
|  | JDSL P | 26 | 0.154 | 0.769 | 0.077 |  | 0.769 | 0.231 |  |
| **LIA** |  | **38** | **0.632** | **0.368** |  |  | **0.763** | **0.237** |  |
|  | LIA O | 20 | 0.950 | 0.050 |  |  | 0.650 | 0.350 |  |
|  | LIA P | 18 | 0.278 | 0.722 |  |  | 0.889 | 0.111 |  |
| **TIA** | TIA O | **5** | **1.000** |  |  |  | **1.000** |  |  |
|  |  |  |  |  |  |  |  |  |  |
| **Haplotype** |  |  |  |  |  |  |  |  |  |
| O |  | 47 | 0.957 | 0.043 |  |  | 0.723 | 0.277 |  |
| P |  | 117 | 0.197 | 0.769 | 0.034 |  | 0.829 | 0.171 |  |
